# Evaluating Cassava Starch Residue as a Maize Substitute in Broiler Chicken Diets

**DOI:** 10.1002/fsn3.70360

**Published:** 2025-06-07

**Authors:** Agnes Osei‐Adjei, Jacob Alhassan Hamidu, Benjamin Adjei‐Mensah, Priscilla Nyameyie Ama Hanson, Goodman Kantanka Sarfo, Armstrong Donkoh

**Affiliations:** ^1^ Department of Animal Science, Faculty of Agriculture Kwame Nkrumah University of Science and Technology Kumasi Ghana; ^2^ Department of Animal Science University of Ghana Legon Ghana

**Keywords:** agro‐waste, cassava by‐product, digestibility, energy resource

## Abstract

A total of 360 1‐week‐old broiler chicks were utilized in a 6‐week experimental study designed to evaluate the effects of varying levels of cassava starch residue (CSR) on growth performance, hematological profiles, nutrient digestibility, and carcass quality in broiler chickens. The chicks were raised in floor pens and divided into six dietary treatments, each comprising 60 birds, with five replicates in a completely randomized design. CSR was incorporated into the diet at inclusion levels of 0%, 20%, 30%, 40%, 50%, and 60%, replacing maize. CSR contains lower quantities of anti‐nutrients such as phytates, oxalates, tannins, saponins, cyanide, and alkaloids, making it more beneficial for farmers to use it in broiler diets without adverse effects. By Day 49, all CSR treatment groups had significantly lower average daily weight gain and final body weight (*p* < 0.05) compared to the control group. The feed conversion ratio (FCR) was significantly improved (*p* < 0.05) in the control, 20%, and 30% CSR groups compared to the 40%, 50%, and 60% CSR groups. Additionally, crude fiber retention was significantly higher (*p* < 0.05) in the CSR treatment diets than in the control diet. Thus, it is suggested that CSR can replace maize up to a 30% inclusion level to optimize feed efficiency and improve carcass yield in broiler chickens.

## Graphical abstract

The study explored the use of cassava starch residue (CSR) as a maize substitute in broiler chicken diets. Excitingly, CSR can replace maize up to 30%, enhancing feed efficiency without negative effects. However, higher CSR levels led to poorer growth performance, making 30% the sweet spot for optimal results. This innovative approach not only offers a sustainable alternative but also supports farmers in reducing feed costs while maintaining high‐quality poultry production.

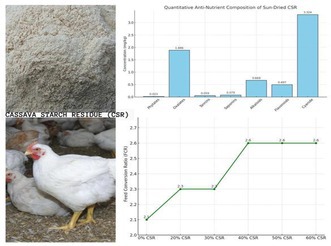

## 1 Introduction

Increasing chicken feed costs have remained a significant issue in underdeveloped nations, where they account for around 65%–75% of overall production costs, whereas in rich nations, they only make up 50%–60% (Mallick et al. 2020; Scott and Vigo 2023). Maize is the main energy source in poultry birds' diets (Dei 2017). However, due to seasonality in production, competition between humans and animals for it as food, and its susceptibility to aflatoxin infection, there is now a significant disparity between the availability and demand of traditional feed supplies for feeding animals, particularly in the tropics. Finding a substitute energy source for poultry has become necessary because of its usage in biodiesel and the demand from domestic businesses and brewers (Akbar et al. 2018). In an effort to address these challenges, scientists are looking for different sources of energy for monogastric animals, particularly pigs and poultry, mainly to lower diet expenses by utilizing the numerous byproducts produced by the food and other agro‐industries (Donkoh and Zanu 2010; Woyengo et al. 2014).

Potentially available non‐conventional feed resources (NCFR) include crop residues, agro‐industrial by‐products, leaf and seed meals, browse foliage, slaughterhouse by‐products, cassava leaf meal, tapioca waste, tea waste, mango seed kernels, and animal organic wastes. Most of these feed materials are low in energy, protein, and minerals and contain high amounts of anti‐nutritional components (Priolo and Ben Salem 2004).

Cassava starch residue (CSR) is a moist solid by‐product generated during cassava starch extraction, accounting for approximately 10%–15% of the initial root weight. It typically contains 53.55% starch and 2.83% ash on a dry matter basis. It contains 53.55% starch, 2.83% ash, 1.98% crude protein (CP), 13.59% crude fiber (CF), and 0.13% ether extract (EE) on a dry matter (DM) basis (Khempaka et al. 2009). In addition, this co‐product also contains a low level of cyanide (< 10 ppm) and does not provide a good substrate for aflatoxin compared to corn and groundnuts (Traiprugsachart et al. 2009; Khempaka et al. 2016). Due to the high starch content of cassava pulp, it can be a valuable alternative energy source for broiler chickens.

In Ghana today, the production of starch for export by some industries is on the rise. Thus, a higher proportion of CSR is produced annually, which warrants proper usage of this cassava by‐product. On the other hand, the high cost of grains, particularly in developing countries, and the increasing competition demand the evaluation of locally available alternative energy feed resources. Previous studies reported that dried CSR can be used in broiler diets at 5%–10% levels without detrimental effects on productive performance. The starch contained in this residue has been reported to be rich in amylopectin and more easily digested by endogenous enzymes than amylose, which is useful for animals (Tester et al. 2004). Furthermore, research has documented the positive effects of CSR's high insoluble fiber content, which enhances growth performance and nutrient digestibility (Jiménez‐Moreno et al. 2013). Increasing the proportions of CSR in the diet of broilers as a replacement for maize beyond the reported 10% may warrant investigation. It was hypothesized that broiler diets incorporated with CSR partially would replace maize as an energy source without having any detrimental effect on the performance of broiler birds. Therefore, this present study investigated the nutrient composition of CSR and its effect on apparent nutrient digestibility, physiological response, growth performance, and carcass quality of broiler chickens.

## 2 Materials and Methods

### 2.1 Ethics and Study Site

The study was carried out at the Poultry Research Unit Department of Animal Science, Kwame Nkrumah University of Science and Technology (KNUST). The study lasted 49 days, beginning on January 24, 2024, and ending on March 16, 2024. The trial was carried out in compliance with the Animal Research Ethics Committee Procedure (AREC) (AREC 2018), KNUST, Kumasi, Ghana.

### 2.2 Source and Preparation of CSR for Analyses

CSR samples were collected from the Amantin Agro Processing Company Limited in the Bono East of Ghana. Based on the sun's intensity and the surroundings' temperature, the freshly harvested CSR was sun‐dried for 5 days on polythene nylon sheets before being ground and stored for analysis and further use.

### 2.3 Proximate and Mineral Composition Analyses of CSR

The DM, EE, ash, CP, and CF contents of the CSR were analyzed according to the procedure of AOAC (2005). The content of nitrogen‐free extract (NFE) was calculated as:

$$\mathrm{NFE}\;\left(\%\right)=\left[\mathrm{DM}-\left(\mathrm{CP}+\mathrm{CF}+\mathrm{EE}+\mathrm{Ash}\right)\right]$$

The metabolizable energy (ME) of the CSR was calculated as follows (Pauzenga 1985):

$$\mathrm{ME}=\;\left[\left(37\times \mathrm{CP}\right)+\left(81.8\times \mathrm{EE}\right)+\left(35\times \mathrm{NFE}\right)\right]$$

After ashing the samples at 550°C to constant weight, the sample was dissolved in deionized water with a few drops of strong hydrochloric acid in a volumetric flask to determine the mineral composition of CSR. A flame photometer (Model 405, Corning, UK) was used to test the amounts of sodium, calcium, and potassium using KCl and NaCl as standards. Iron, copper, and magnesium were measured using Buck 600 AAS atomic absorption spectrophotometry. The Spectric 21D digital spectrometer (Milton Roy, Illinois, US) was used to measure the amounts of manganese and phosphorus. All analyses were done in triplicate.

### 2.4 Antinutrients Determination

Antinutrients, including flavonoids, cyanide, tannin, saponin, phytate, and oxalate in CSR, were determined. The titration method as defined by Siger et al. (2012) was used to quantify the amount of oxalates in the sample. The procedure of Baltha and Cereda (2006) was followed to determine cyanide content using KCN or acetone‐cyanidin as the pattern. Additionally, phytate was determined following the methods of Abudabos (2012). Tannins were determined by using the Folin‐Denis spectrophotometric method, and flavonoids were determined following the method of Boham and Kocipai (1994) and Ejikeme et al. (2014).

### 2.5 Experimental Birds and Management

Three hundred and sixty unsexed Ross 308 broiler day‐old chicks were obtained from a reputable day‐old chicks importer. After 7 days of brooding (fed pre‐starter diet, 22% CP, 3150 kcal/kg ME), the chicks were weighed individually and randomly placed in their respective 6 treatments with 5 replicates each and 60 birds per treatment group in a completely randomized design. The floor of the pens was covered with wood shavings of 4 cm thickness with a stocking density of 10 birds/m^2^. The experiment lasted for 6 weeks. The dietary treatments comprised a standard diet (control, 0% CSR) and a standard diet with CSR incorporation at 20%, 30%, 40%, 50%, and 60%. The levels of inclusion of CSR in this study were based on earlier works of Khempaka et al. (2009) and Kumsri et al. (2009) to ascertain whether higher levels of the test material can impact the performance of broiler chickens. Birds were fed these diets in mash form, with water available *ad libitum*. They were kept on a lighting schedule of 12 h of light, maintained at an average temperature of 27.8°C and a relative humidity of 83%. Prophylaxis treatment and vaccination schedules were followed as recommended by the Veterinary Service Directorate. Experimental diets were formulated in accordance with the guidelines established by the National Research Council and Subcommittee on Poultry Nutrition (1994), taking into consideration the specific nutritional requirements for broiler starter and finisher phases. The ingredients and chemical composition of the diets are shown in Table 1.

**TABLE 1:** Ingredients and nutritional composition of the experimental diets (as‐fed basis).

	Starter diet						Finisher diet					
	0% CSR	20% CSR	30% CSR	40% CSR	50% CSR	60% CSR	0% CSR	20% CSR	30% CSR	40% CSR	50% CSR	60% CSR
Ingredients												
Maize	55.54	44.43	38.88	33.32	27.77	16.66	65.55	52.44	45.72	39.33	32.78	19.67
CSR		11.11	16.66	22.22	27.77	38.88		13.11	19.67	26.22	32.78	45.72
Soya	20.4	20.45	22.85	21.6	22.5	23.3	14.63	13.07	14	14.14	14.3	15.39
Fish	10	11.50	12.20	12.50	12.50	12.25	10.74	12.25	13. o4	13.95	14.87	14.92
Con	3	3	3	3	3	3	—	—	—	—	—	—
WB	8	6.2	5.25	4.2	3.3	2.5	5	4.5	3.5	2.5	1.7	0.7
Lysine	0.3	0.3	0.3	0.3	0.3	0.3	0.3	0.3	0.3	0.3	0.3	0.3
Methionine	0.3	0.3	0.3	0.3	0.3	0.3	0.2	0.2	0.2	0.2	0.2	0.2
Toxin	0.2	0.2	0.2	0.2	0.2	0.2	0.2	0.2	0.2	0.2	0.2	0.2
Salt	0.1	0.1	0.1	0.1	0.1	0.1	0.1	0.1	0.1	0.1	0.1	0.1
Oyster shells	1.3	1.3	1.3	1.3	1.3	1.3	2	2	2	2	2	2
Vitamin Premix*	0.2	0.2	0.2	0.2	0.2	0.2	0.3	0.3	0.3	0.3	0.3	0.3
DICAL/PHOS	0.3	0.3	0.3	0.3	0.3	0.3	0.3	0.3	0.3	0.3	0.3	0.3
Palm oil	0.46	0.46	0.46	0.46	0.46	0.46	0.69	0.69	0.69	0.69	0.69	0.69
Total	100.00	100.00	100.00	100.00	100.00	100.00	100.00	100.00	100.00	100.00	100.00	100.00
Determined nutrients (%)												
Ash	11.75	13.82	13.70	14.41	16.01	16.05	11.26	11.52	11.89	11.56	12.66	12.75
Crude protein	22.85	22.69	22.50	22.18	22.16	21.98	17.69	17.39	17.30	17.23	17.21	17.21
Crude fat	4.82	4.92	4.86	6.94	7.22	7.58	8.41	8.49	8.54	8.32	8.33	8.52
Crude fiber	2.85	2.95	3.26	3.31	3.39	5.61	7.88	8.24	8.08	8.3	8.5	8.6
Lysine	1.38	1.38	1.37	1.35	1.35	1.35	1.12	1.12	1.10	1.00	1.00	1.00
Methionine	0.62	0.62	0.61	0.60	0.60	0.60	0.58	0.58	0.57	0.55	0.55	0.55
Calcium	1.02	1.02	1.00	0.95	0.95	0.95	0.93	0.93	0.92	0.91	0.91	0.90
Phosphorus	0.71	0.71	0.7	0.69	0.69	0.69	0.62	0.62	0.59	0.58	0.58	0.58
M.E. (kcal/kg)*	2845.52	2797.38	2792.45	2837.35	2800.36	2738.10	2875.82	2864.61	2858.37	2873.1	2810.91	2819.46

*Note:* 0% CSR (control, standard diet), 20% CSR, 30% CSR, 40% CSR, 50% CSR, and 60% CSR. M.E. (kcal/kg)* = (37* Protein) + (81.8* fat) + (35* NFE).

Abbreviations: Con = concentrate, CSR = cassava starch residue, M.E. = metabolizable energy, NFE = nitrogen‐free extra, WB = wheat bran.

### 2.6 Growth Parameters

Records of daily feed consumption and weekly body weight of the birds were taken. Body weight gain (BWG) was calculated by deducting the previous week's initial body weight from the final body weight of the current week. The feed conversion ratio was computed by dividing the average feed consumed per bird by the average weight gain after mortality was corrected.

### 2.7 Metabolic Cage Trial

At the end of 21 and 42 days, eight birds per treatment were randomly selected from the six treatment groups for a metabolic cage study which lasted for 5 days after an adjustment period of 2 days. A known quantity of feed was given, and feces voided were daily collected for 5 days. Dropping trays were placed under each cage for excreta collection. Records of feed consumption within the 5 days were taken. The fecal samples collected from each replicate were sprayed with 5% HCl and oven‐dried to remove the moisture content (Donovan et al. 2017) to bring the feces to a constant weight after the feathers and contaminants were removed. The total collection of feces was pooled, weighed, and ground, and representative samples were taken to the laboratory for chemical analysis according to standard methods (AOAC 2005). The proximate composition of the diets and excreta results was used to calculate the digestibility as outlined in Onimisi et al. (2008) using the formula:

$$\begin{array}{c} \text{Apparent Nutrient Digestibilty}\;\left(\%\right)\\ =\left[\frac{\left(\text{Nutrient in feed}-\text{Nutrient in feces}\right)}{\text{Nutrient in fedd}}\right]\times 100 \end{array}$$

### 2.8 Blood Collection and Evaluation

Using a sterile syringe and needle, 5 mL of blood samples were randomly taken from 4 birds per treatment from the left wing for hematological examination at the end of the starter and finisher phases. The blood samples were kept in ethylene‐diamine tetra‐acetic acid (EDTA) tubes for analysis. The counts and concentrations of platelets, mean corpuscular hemoglobin (MCH), white blood cells (WBC), hemoglobin, red blood cells (RBC), hematocrit, and mean corpuscular volume (MCV) were analyzed using a hematoanalyzer for chickens.

### 2.9 Carcass Evaluation

At the end of the experiment, two birds were randomly selected from each replicate, weighed, and humanely sacrificed for carcass evaluation. The bled weight of the birds was also recorded after bleeding for about 5–10 min after slaughtering. The birds were then dissected to remove the internal organs and then wet‐plucked. Carcass cut parts and internal organs were weighed and expressed as a percentage of the live weight of the birds. The breast muscles were fragmented into minor and major portions and weighed separately using a 5000 g sensitive electronic balance and expressed as a percentage of the live weight following the procedure of Koranteng et al. (2022).

### 2.10 Statistical Analysis

Data obtained were subjected to analysis of variance (ANOVA) using the general linear model, Minitab (version 19.0) following the model: yij = m + tj + eij, where m is the general mean, *t* is the treatment effect, and *e* is random error. Values obtained are presented as mean and standard errors of the means. The Tukey post hoc test was used to compare and separate the means at a significance level of 5%. The linear and quadratic treatment effects were determined using orthogonal polynomial contrasts in R (version 4.3).

## 3 Result and Discussion

### 3.1 Chemical Analysis

The results of the proximate analyses of the sun‐dried CSR are presented in Table 2. Additionally, information about maize is supplied for comparative reasons. The test ingredient had a moisture content (of 10.95%). The value fell within the 10%–13% range reported by Ukita et al. (2006) and that of maize Dudley‐Cash (2014). The relatively low moisture content of the residue after sun drying suggests a long shelf life of the dried residue. Since CSR is lighter and fluffier than cassava chips, it would require less drying time (Dzisi and Wirth 1994).

**TABLE 2:** Proximate analysis (%) of sun‐dried CSR and maize (as fed).

Parameter (g kg^−1^ DM)	CSR^a^	Maize^b^
Moisture	8.34	10.20
Crude protein	2.52	8.92
Crude fat	5.14	4.48
Crude fiber	4.46	1.93
Ash content	2.50	1.90
Dry matter	91.66	88.75
Nitrogen free extract	73.42	82.77
Metabolizable energy (kcal/kg)	3035.68	3415.50
NDF	17.27	9.6
ADF	7.72	2.8
Hemicellulose	9.55	6.8

Abbreviations: ADF = acid detergent fiber, NFE = nitrogen‐free extract.

^a^ Values based on triplicate samples.

^b^ Values reported by Dudley‐Cash (2014).

As expected, the DM content of the residue was high (91.66%) compared to that of maize, which is 88.75% as reported by Dudley‐Cash (2014). The DM content of cassava could vary depending on the age of the plant, season, and location, and with temperature factors related to location (Enesi et al. 2022). The CP of 2.52% for the residue was greater than the 1.55% stated by Suksombat et al. (2006) but lower than that of maize. The difference in the CSR CP values might be a result of the difference in soil composition and geographical location. Iyayi and Losel (2001) findings also agree with the value obtained in this study. The lower protein content of the residue might not enhance the growth and maintenance of tissue if it is not supplemented. The following were the CF fractions obtained for CSR in this study: hemicellulose (9.55%), acid detergent fiber (ADF) (7.72%), and neutral detergent fiber (NDF) (17.27%). For maize, Stein et al. (2016) and Dudley‐Cash (2014) found that the comparable values for NDF, ADF, and hemicellulose were 10.27%, 2.90%, and 7.30%, respectively. The proportion of NDF and hemicellulose reported in this current study is greater than those for maize. This implies that there is a possible greater proportion of slower‐digesting fibrous material in CSR relative to maize. All of the fibrous components in feeds are measured by NDF, which has an inverse relationship with the energy content (Mertens and Dado 1993; Manthey et al. 2016). Fiber, as a nutrient, is harder to digest by monogastric animals than NFE and determines dietary nutritive value because it influences the proportion of chemically available nutrients that can be utilized by the animals. This is a result of the low digestibility of fibers. The CF value of 4.46% for the residue CSR was lower than the 7.75% reported by Suksombat et al. (2006) but higher compared to that of maize, which is 1.93% reported by Dudley‐Cash (2014). Fiber is considered a significant component of the diet since it can increase stool bulk and decrease the time waste materials spend in the gut (Mudgil and Barak 2013).

The crude fat in the CSR (5.14%) was much higher than the value recorded by Khempaka et al. (2009) and also greater than that of maize, as reported by Dudley‐Cash (2014), which would contribute to the energy supply using this feedstuff. Fat and oils are the most abundant lipids found in nature, which also contain the vital fat‐soluble vitamins (A, D, E, and K). The NFE value of CSR (73.42%) showed that the residue contains a considerable level of carbohydrates that could qualify it as a good candidate for microbial fermentation, as the soluble carbohydrates it contains could serve as suitable substrates for the growth of the microbial inoculums (Aro et al. 2010) although lower than that of maize. The energy value (3035.68 kcal/kg) of sun‐dried CSR was very close to that of maize (3434 kcal/kg) as reported by Dudley‐Cash (2014). The test ingredient can therefore be described as a potential energy feedstuff and could contribute to the energy level in the diets of poultry birds to meet their energy requirement.

### 3.2 Mineral‐Elements Composition of CSR and Maize

Table 3 shows the results of the mineral elements examined in this investigation. The amount of ash in a sample often indicates its inorganic mineral composition (FAO 2006). The ash level of the CSR in this investigation was greater than that of maize (Table 1), which may indicate that the test component contains more necessary inorganic minerals. The level of calcium obtained in this study for CSR was greater than the value (0.03%) reported by Dudley‐Cash (2014) for maize, while that of magnesium was the same. Potassium, sodium, and phosphorus levels in the CSR were lower compared to those of maize for the macro‐elements. However, calcium and phosphorus content in the CSR (0.363% and 0.078% respectively) fell within the range of 0.19%–1.76%, and 0.06%–1.52%, respectively, reported by Gleadow et al. (2009). Sarkiyayi and Agar (2010) also reported that sweet and bitter varieties of cassava have 0.33% and 0.30% of calcium, respectively. The magnesium content was 0.121%. Gleadow et al. (2009) reported a much lower value of 0.03%–0.08%. Feed formulations for animal diets should include macro‐mineral components, including calcium, phosphorus, and magnesium as essential nutrients (Li and Patience 2017). They are constituents of body fluids and structures (electrolytes) and participate in the biochemical processes that take place within the body of an animal. They are also important constituents of bones and are involved in blood clotting. The iron content was much lower than the values of 149–300 mg/kg reported by Sarkiyayi and Agar (2010). The level of iron (7.87 mg/kg) in this current study fell within the range of values reported by Sarkiyayi and Agar (2010). Iron is very important because of its occurrence in many proteins such as hemoglobin, myoglobin, and cytochromes (Robert et al. 2006). Its deficiency causes nutritional anemia and a decrease in hemoglobin levels. The level of potassium in this study, that is, 0.0030%, was less than that reported by Adedokun et al. (2017). Potassium is involved in membrane function and carbohydrate metabolism, and potassium in combination with sodium chloride functions in maintaining acid–base and ionic balance of body fluids (Olomu 1995; Robert et al. 2006).

**TABLE 3:** Determined mineral‐elements composition of CSR and maize.

Minerals	CSR^a^	Maize^b^
Calcium (g kg^−1^ DM)	0.363	0.030
Phosphorous (g kg^−1^ DM)	0.078	0.280
Potassium (g kg^−1^ DM)	0.030	0.33
Magnesium (g kg^−1^ DM)	0.121	0.120
Sodium (g kg^−1^ DM)	0.007	0.020
Iron (mg kg^−1^)	7.867	0.029
Manganese (mg kg^−1^)	1.263	0.007

Abbreviation: CSR = cassava starch residue.

^a^ Values based on triplicate samples.

^b^ Values reported by Dudley‐Cash (2014), Stein et al. (2016).

### 3.3 Qualitative and Quantitative Anti‐Nutrient Composition of the CSR

The qualitative and quantitative anti‐nutrient composition of the CSR is presented in Table 4. Qualitatively, phytates, oxalates, tannins, saponins, and alkaloids were present in the CSR; however, the quantities present were lower than what Khang (2004) and Sarkiyayi and Agar (2010) reported. More importantly, the levels observed suggested that the residue has lower levels of these anti‐nutrients and will make it more advantageous for farmers to use them in broiler diets without any detrimental effect on the growth of the birds. It is therefore unlikely that their concentrations in the dried CSR would compromise its nutritional potential. Mathams and Sutherland (1952) stated that for plants to be potentially unsafe, they should contain at least 10% (100,000 ppm) oxalate on a dry weight basis. The tannin content in the CSR sample, when compared to the typical concentrations found in various legumes as reported by D'Mello (2000), is classified as very low to moderate. Consequently, it can be concluded that CSR contains very low levels of tannic acid. Additionally, the cyanide level in sundried CSR was measured at 32.4 mg/kg, which is below the harmful threshold of 50 mg/kg suggested for animals (Canadian Food Inspection Agency CFIA 2025). Cyanide decreases the quantity of hemoglobin in the blood and impedes the effective transport of oxygen and carbohydrates due to its high attraction to minerals like iron. By preventing the intra‐thyroidal absorption of iodine, it also lowers the growth rate by increasing the production of thyroid‐stimulating hormone (TSH), which in turn lowers the amount of thyroxin, which is necessary for growth (Tewe 1992).

**TABLE 4:** Qualitative and quantitative anti‐nutrient composition of sun‐dried CSR.

Parameters	Qualitative	Quantitative value (mg/kg)
Phytates	+	0.023
Oxalates	+	1.886
Tannins	+	0.059
Saponins	+	0.079
Alkaloids	+	0.669
Flavonoids	+	0.497
Cyanide	+	3.3240

*Note:* + = presence.

### 3.4 Growth Parameters

The growth performance results of broilers fed different quantities of CSR are shown in Table 5. Body weight, daily weight gain, final body weight, and FCR all showed significant differences (*p* < 0.05). The final body weight values ranged from 2000.0 g (60%) to 2512.5 g (Control) and 1229.51 g (60%) to 1269.31 g (Control) at 28 days. There was a linear effect on body weight at Day 28 (*p* = 0.008). This showed that the treated diets met the nutrient requirement for raising broiler chickens (Yan et al. 2010). Feed intake did not differ significantly among treatments; however, the FCR of birds was significantly better in the control diet compared to the CSR‐based diets (*p* = 0.0001) at Day 49. Furthermore, the FCR of 20% and 30% CSR was better than 40%–60% incorporation of CSR (*p* = 0.0001). BWG was significantly superior in the control diet, followed by 20%–30% CSR‐based diets to the 40%–60% CSR‐based diets. These trends were also observed in the overall performance (7–49 days). There were quadratic and cubic effects on BWG and FCR (*p* = 0.0001; *p* = 0.038). Interestingly, higher inclusion levels of CSR in the diet led to poor feed utilization, suggesting that CSR can successfully replace maize up to 30% of the diet of broiler chickens. A study by da Silva et al. (2019) showed that the incorporation of dry residue of cassava linearly increased the FCR of the birds at a higher inclusion rate of 10% when Cobb 500 broiler chicks were reared for 42 days. In another study, the inclusion of dry cassava factory residue at 10% in the diet of slow‐growing broilers negatively influenced weight gain and feed intake at Day 49 (Picoli et al. 2014). Additionally, Almeida et al. (2024) observed a quadratic increasing effect of dry cassava residue on feed intake and weight gain at Day 42 when slow‐growing broilers received a diet of up to 10%. It is interesting to note that Chang'a et al. (2020) found that adding dry cassava residue to broiler diets reduced body weight and raised FCR as levels increased (25%–75%). The rising CF content in the diet as CSR levels increase may be the cause of the declining trend in the weight gain of birds. Furthermore, growth performance could have been negatively impacted by the concentration of resistant starch and non‐starch polysaccharides (NSPs) in the starch residue obtained from cassava roots (George and Sese 2012). Given that avian species are unable to effectively digest these components, their presence can result in impaired absorption of starch‐ and sucrose‐derived glycosides, modifications to intestinal motility, and ultimately a reduction in nutrient availability and the efficiency of nutrient absorption (Picoli et al. 2014).

**TABLE 5:** Growth performance parameters of broiler chickens fed control and CSR‐based diets at different inclusion levels.

Parameters	Dietary treatments							*p*			
	0% CSR	20% CSR	30% CSR	40% CSR	50% CSR	60% CSR	SEM^1^	Main effect^2^	Linear effect^3^	Quadratic effect^4^	Cubic effect^5^
7–28 days											
Initial body weight (g)	250.54	257.60	251.25	250.08	251.92	249.42	4.65	0.899	0.395	0.580	0.741
Daily feed intake (g)	85.53	87.80	87.28	87.44	85.90	85.74	1.37	0.415	0.0447	0.977	0.674
Daily weight gain (g)	46.31	45.94	45.34	45.05	44.61	44.55	2.02	0.617	0.703	0.104	0.739
FCR (g/g)	1.85	1.91	1.93	1.94	1.93	1.92	0.11	0.687	0.801	0.118	0.700
Body weight (g)	1269.31^a^	1268.37^a^	1248.75^b^	1241.07^bc^	1233.39^cd^	1229.51^d^	34.7	0.0001	0.008	0.620	0.462
29–49 days											
Daily feed intake (g)	135.70	134.75	137.77	139.91	135.62	133.78	7.00	0.957	0.784	0.642	0.462
Daily weight gain (g)	59.20^a^	49.72^b^	49.46^b^	38.70^c^	38.89^c^	36.69^c^	1.89	0.0001	0.685	0.0001	0.0002
FCR (g/g)	2.3^c^	2.7^b^	2.8^b^	3.6^a^	3.5^a^	3.6^a^	0.32	0.0001	0.931	0.0001	0.001
Body Weight (g)	2512.5^a^	2312.5^b^	2287.5^b^	2053.8^c^	2050.0^c^	2000.0^c^	52.9	0.0001	0.674	0.0001	0.0001
7–49 days											
Total feed intake (g)	4645.8	4673.4	4726.0	4774.0	4652.0	4609.9	154	0905	0.512	0.660	0.436
Total weight gain (g)	2262.0^a^	2057.9^b^	2036.2^b^	1803.7^c^	1798.1^c^	1750.6^c^	52.8	0.0001	0.619	0.0001	0.0001
FCR	2.1^c^	2.3^b^	2.3^b^	2.6^a^	2.6^a^	2.6^a^	0.088	0.0001	0.375	0.0001	0.038

*Note:* 0% CSR (control, standard diet), 20% CSR, 30% CSR, 40% CSR, 50% CSR, and 60% CSR.

Abbreviations: CSR = cassava starch residue, FCR = feed conversion ratio. a‐d Different superscript letters indicate significant difference between means within the same rows (*p* < 0.05).

^1^ Pooled standard error of means.

^2^ Statistical comparison of means between all dietary treatments.

^3^ Linear contrast between inclusion levels.

^4^ Quadratic contrast between inclusion levels.

^5^ Cubic contrast between inclusion levels.

### 3.5 Carcass Parameters

The carcass parameters of broiler chickens fed experimental diets are shown in Table 6. There were significant differences (*p* < 0.05) for live weight and abdominal fat. The live weight of birds in the control group was significantly higher than that in the CSR‐based groups (*p* = 0.0001), which in turn had quadratic and cubic effects (*p* = 0.0009; *p* = 0.0001). Birds that received the 60% CSR treatment diet had the lowest value (0.75%) of abdominal fat compared with that of the control and the rest of the treatments (*p* = 0.022). Moreover, there was a cubic effect on abdominal fat (*p* = 0.008). The positive comparison of the treated feed with the control showed that birds fed with the treated diet had less fat deposition than those fed the control diet. Dressed and breast yields did not differ significantly among treatments. On the contrary, Picoli et al. (2014) observed a significant impact of cassava starch factory residue on breast yield but did not affect abdominal fat at the inclusion levels of 2%–10% in ISA Label JA57 slow‐growing broilers. There was a significant decrease in dressed weight and breast weight when broilers were fed dry cassava residue at 75% for 35 days. The dressed weights of all the diets supported the assertion that at various levels of inclusion of CSR as a replacement for maize, the birds can perform well without any deleterious effect on performance since dressed weight is suggested to be a relative value of dressed saleable carcass for maximum profit in broiler chickens (Aboki et al. 2013). The organ proportion of broiler chickens fed an experimental diet shows that there were no statistical differences observed in full GIT, liver, full gizzard, empty gizzard, and full viscera spleen (*p* > 0.05). The current finding disagrees with that of Picoli et al. (2014) who observed significant changes in gastrointestinal organs when cassava starch factory residue was fed to a slow‐growing broiler at low levels (2%–10%). The differences observed in the reported literature can be due to the levels of inclusion of the cassava by‐products.

**TABLE 6:** Carcass parameters of broiler chickens fed control and CSR‐based diets at different inclusion levels.

Parameters	Dietary treatments							*p*			
	0% CSR	20% CSR	30% CSR	40% CSR	50% CSR	60% CSR	SEM^1^	Main effect^2^	Linear effect^3^	Quadratic effect^4^	Cubic effect^5^
Live weight (g)	2512.5^a^	2312.5^b^	2287.5^b^	2053.8^c^	2050.0^c^	2000.0^c^	52.9	0.0001	0.674	0.0009	0.0001
Bled weight (%)	95.53	95.66	95.08	94.94	93.30	94.99	0.73	0.056	0.776	0.561	0.014
Defeathered weight (%)	84.33	85.58	82.73	85.11	79.58	85.01	2.90	0.341	0.776	0.235	0.235
Dressed weight (%)	84.46	81.84	76.33	80.64	75.67	82.54	5.14	0.481	0.391	0.172	0.832
Full GIT weight (%)	13.73	12.01	13.67	13.85	14.85	13.23	1.19	0.345	0.256	0.110	0.509
Liver weight (%)	2.14	2.21	2.024	1.89	2.45	2.31	0.22	0.197	0.436	0.952	0.041
Full gizzard weight (%)	3.33	3.14	2.94	3.47	3.18	3.06	0.33	0.676	0.613	0.913	0.687
Empty gizzard Weight (%)	1.59	1.52	1.47	1.65	1.59	1.43	0.16	0.766	0.820	0.780	0.577
Full viscera weight (%)	7.56	8.60	7.56	9.99	7.20	7.24	2.22	0.786	0.504	0.852	0.702
Major breast weight (%)	18.11	17.19	16.42	15.88	14.42	16.56	1.85	0.500	0.7509	0.079	0.597
Minor breast weight (%)	4.26	4.16	3.81	3.59	3.39	4.10	0.57	0.604	0.744	0.106	0.905
Full breast weight (%)	22.97	21.42	20.26	19.55	17.83	20.65	3.41	0.468	0.618	0.065	0.997
Spleen weight (%)	0.14	0.16	0.12	0.06	0.11	0.13	0.046	0.469	0.972	0.089	0.478
Abdominal fat (%)	1.17^a^	0.99^ab^	1.03^ab^	0.85^ab^	0.78^ab^	0.75^b^	0.12	0.022	0.149	0.050	0.008

*Note:* 0% CSR (control, standard diet), 20% CSR, 30% CSR, 40% CSR, 50% CSR, and 60% CSR.

Abbreviation: CSR = cassava starch residue. a‐c Different superscript letters indicate significant difference between means within the same row (*p* < 0.05).

^1^ Pooled standard error of means.

^2^ Statistical comparison of means between all dietary treatments.

^3^ Linear contrast between inclusion levels.

^4^ Quadratic contrast between inclusion levels.

^5^ Cubic contrast between inclusion levels.

### 3.6 Apparent Nutrient Digestibility

Table 7 shows the nutrient digestibility of broiler chickens fed a diet containing CSR. Crude fat digestibility was unaffected by the dietary interventions throughout the starter phase (*p* > 0.05). The groups given the CSR in their diets retained a greater (*p* = 0.0001) ash level. The highest value for crude protein digestibility was observed in 60% CSR and the lowest values were recorded in control, 30% CSR, and 50% CSR (*p* = 0.003). For crude fiber, the highest values were recorded in control and 30% CSR (89.778% and 89.655%, respectively) (*p* = 0.0001). The highest ME value was recorded in the control diet and the lowest value in 20% CSR (*p* = 0.0001). For the finisher phase, crude fiber content and ME differed significantly (*p* = 0.002; *p* = 0.023) respectively. The highest crude fiber value was recorded in the 60% CSR diet and the lowest value was in the control diet. For ME, 30% CSR had the highest ME value and 60% CSR had the lowest ME value. This implies that as the levels of CSR increased from 30% in the diet, less energy was retained by the birds in these groups. However, crude fiber utilization was improved as the proportions of CSR increased in the diet. The observed differences in nutrient digestibility between the dietary treatments could be attributed to several factors. The inclusion of CSR in the diet may have altered the composition and properties of the feed, thereby affecting its digestibility. Processed agro‐industrial by‐products have also been shown to be a viable ingredient in broiler diets, with no negative impact on growth performance or feed efficiency (El‐Deek et al. 2020; Georganas et al. 2023). However, the processing techniques employed in agro‐industrial by‐products may not always improve their feeding value for broiler chickens (Malenica et al. 2022). The effects of such processing can be variable and depend on factors such as the specific ingredients used, processing conditions, and the characteristics of the final product (Chrystal et al. 2020). While some studies have shown the benefits of using processed agro‐industrial by‐products in broiler diets (Sugiharto et al. 2018) the impact on nutrient digestibility and overall performance can be inconsistent.

**TABLE 7:** Apparent nutrient digestibility of broiler chickens fed control and CSR‐based diets at different inclusion levels during starter and finisher phases.

Parameters	Dietary treatments							*p*			
	0% CSR	20% CSR	30% CSR	40% CSR	50% CSR	60% CSR	SEM^1^	Main effect^2^	Linear effect^3^	Quadratic effect^4^	Cubic effect^5^
Starter phase											
Ash content (%)	74.50^b^	88.26^a^	87.38^a^	86.00^a^	86.25^a^	86.25^a^	0.800	0.0001	0.0001	0.0002	0.0001
Crude protein (%)	75.64^b^	78.27^ab^	75.52^b^	77.33^ab^	75.25^b^	79.68^a^	0.980	0.003	0.443	0.472	0.005
Crude fat (%)	97.75	97.25	97.25	97.50	96.25	96.75	0.493	0.178	0.886	0.7579	0.216
Crude fiber (%)	89.78^a^	88.53^ab^	89.83^a^	89.66^ab^	88.00^bc^	86.82^c^	0.538	0.0001	0.1780	0.2348	0.0003
ME (kcal/kg)	1956.50^a^	1704.20^c^	1783.23^bc^	1834.20^b^	1847.30^b^	1831.80^b^	28.4	0.0001	0.110	0.84016	0.005
Finisher phase											
Ash content (%)	67.65	66.41	68.74	67.79	67.62	68.44	2.86	0.974	0.765	0.651	0.774
Crude protein (%)	83.52	84.90	81.83	80.74	84.57	82.83	2.13	0.365	0.913	0.243	0.214
Crude fat (%)	90.44	91.43	92.22	92.05	90.44	93.15	1.05	0.149	0.676	0.484	0.697
Crude fiber (%)	80.52^b^	88.95^ab^	86.73^ab^	91.85^a^	92.90^a^	93.99^a^	2.94	0.002	0.101	0.082	0.001
ME (kcal/kg)	2354.10^ab^	23333.30^ab^	2427.60^a^	2321.10^ab^	2279.90^ab^	2240.30^b^	49.0	0.023	0.144	0.550	0.009

*Note:* 0% CSR (control, standard diet), 20% CSR, 30% CSR, 40% CSR, 50% CSR, and 60% CSR.

Abbreviation: CSR = cassava starch residue. a‐c Different superscript letters indicate signifcant difference between means within the same row (*p* < 0.05).

^1^ Pooled standard error of mean.

^2^ Statistical comparison of means between all dietary treatments.

^3^ Linear contrast between inclusion levels.

^4^ Quadratic contrast between inclusion levels.

^5^ Cubic contrast between inclusion levels.

### 3.7 Blood Parameters

Table 8 shows the impact of dietary CSR on the experimental birds' hematological parameters. White blood cell (WBC) levels after 28 days showed a quadratic impact (*p* = 0.0431), although there was no significant difference (*p* > 0.05) between treatment diets during the starting phase. Some blood indicators showed significant differences (*p* < 0.05) between the treatment diets during the finisher phase. A marked difference (*p* < 0.05) was observed in WBC and ranged from 186.72 to 210.40, with control having the highest WBC count and T2 having the lowest count. Additionally, there was a linear effect (*p* = 0.000875) on the WBC count. For percent lymphocyte (LYM), CSR levels of 30% and 40% differed significantly from the control group (*p* = 0.0001). The lowest value (87.5%) was recorded from the control group, whereas the highest values were recorded from CSR levels of 30% and 40% (95.35% and 94.23%, respectively). There were also linear and quadratic effects on the percent lymphocytes (*p* = 0.0002; *p* = 0.013).

**TABLE 8:** Hematological indices at Days 21 and 49 of broiler chickens fed control and CSR‐based diets at different inclusion levels.

Parameters	Dietary treatments							*p*			
	Control	20% CSR	30% CSR	40% CSR	50% CSR	60% CSR	SEM^1^	Main effect^2^	Linear effect^3^	Quadratic effect^4^	Cubic effect^5^
Day 21											
WBC (×10^6^/μL)	191.45	194.10	198.0	201.18	213.20	198.35	8.94	0.257	0.9974	0.0431	0.462
RBC (×10^3^/μL)	2.24	2.30	2.28	2.28	2.39	2.29	0.13	0.908	0.833	0.526	0.535
HGB (g/dL)	8.375	8.85	8.15	8.66	8.85	8.68	0.49	0.684	0.853	0.878	0.151
HCT (%)	32.525	33.48	31.73	32.60	33.80	32.47	1.58	0.805	0.0891	0.985	0.336
MCV (fL)	145.35	145.73	139.43	142.80	117.0	142.05	15.2	0.427	0.827	0.187	0.570
MCH (pg)	37.30	38.50	35.80	37.68	37.15	37.90	0.90	0.116	0.9666	0.316	0.042
MCHC (g/dL)	25.68	26.43	25.70	26.40	26.68	26.70	0.57	0.309	0.8903	0.273	0.043
PLT (×10^6^/μL)	64.8	63.0	51.5	61.8	44.00	67.0	32.9	0.978	0.849	0.653	0.928
LYM (%)	64.65	63.95	63.7	63.72	60.4	64.10	13.4	0.175	0.8536	0.132	0.857
NEUT (%)	2.65	3.10	3.38	3.35	4.84	3.300	1.32	0.684	0.901	0.229	0.548
Day 49											
WBC (×10^6^/μL)	210.40^a^	187.50^b^	186.72^b^	203.73^ab^	203.60^ab^	190.75^b^	5.37	0.001	0.0008	0.626	0.105
RBC (×10^3^/μL)	2.44	2.50	2.49	2.28	2.45	2.64	0.17	0.437	0.539	0.707	0.264
HGB (g/dL)	9.10	8.73	9.30	8.36	8.88	9.70	0.71	0.525	0.4025	0.808	0.667
HCT (%)	34.63	32.60	33.73	31.23	33.05	35.38	2.14	0.469	0.199	0.484	0.673
MCV (%)	141.82	132.47	135.25	137.45	135.32	133.93	3.64	0.204	0.075	0.639	0.077
MCH (pg)	37.23	35.45	37.18	36.85	36.23	37.00	1.08	0.552	0.242	0.663	0.235
MCHC (g/dL)	26.23	26.78	27.50	26.83	26.80	27.60	0.49	0.097	0.402	0.163	0.478
PLT (×10^6^/μL)	42.00	31.30	66.00	34.25	50.80	38.80	18.6	0.474	0.918	0.523	0.324
LYM (%)	57.5^b^	62.38^ab^	65.35^a^	64.23^a^	58.7^b^	61.08^ab^	14.1	0.0001	0.0002	0.013	0.141
NEUT (%)	2.63	2.750	3.88	4.17	2.43	3.40	2.20	0.332	0.130	0.409	0.605

*Note:* 0% CSR (control, standard diet), 20% CSR, 30% CSR, 40% CSR, 50% CSR, and 60% CSR.

Abbreviations: CSR = cassava starch residue, HCT = hematocrit, HGB = hemoglobin, LYM = lymphocytes, MCHC = mean corpuscular hemoglobin concentration, MCH = mean corpuscular hemoglobin, MCV = mean corpuscular volume, NEUT = neutrophils, PLT = platelet, RBC = red blood cell, WBC = white blood cells. a‐b Different superscript letters indicate significant difference between means within the same row (P < 0.05).

^1^ Pooled standard error of means.

^2^ Statistical comparison of means between all dietary treatments.

^3^ Linear contrast between inclusion levels.

^4^ Quadratic contrast between inclusion levels.

^5^ Cubic contrast between inclusion levels.

White blood cells provide antibodies for the immune system and protect the body from invasion by foreign substances. The control group and the treatment groups differed significantly from one another. According to Jain (1993), all of the inclusion level values for WBC were below the typical range (120–300 × 10^3^/μL). This suggests that CSR affects the birds' immunological state, which is an inherent body defense system that maximizes performance under stressful circumstances (Onunkwo et al. 2022). According to Soetan and Oyewole (2009), animals with high WBC levels are capable of producing antibodies and exhibiting a high level of disease resistance. Birds with low WBC are more likely to contract diseases, whereas those with higher WBC can develop antibodies as part of humoral immunity and have greater disease resistance (Soetan and Oyewole 2009).

The current findings on the values of hemoglobin align with the values of 7.04–13.0 g/dL reported by Putriani et al. (2012) and that of 8.09–10.17 g/dL reported by Onu et al. (2024). One important metric for assessing circulating erythrocytes and a key factor in the diagnosis of anemia is hemoglobin (Peters et al. 2011). Additionally, it is a helpful indicator of the ability of the bone marrow to generate red blood cells in animals (Chineke et al. 2006).

The results suggest that the incorporation of CSR in broiler diets at an inclusion level of up to 30% enhances feed utilization and improves the carcass yield of broiler chickens. Furthermore, the inclusion of CSR did not adversely affect the health benefits of the broilers; rather, it appeared to positively influence the birds' immune status, which functions as an intrinsic body defense system. Alternative research approaches, including enzyme supplementation to break down the high fiber content of CSR, are essential and warrant further investigation for its usage in the commercial poultry feed industry.

## Author Contributions

**Agnes Osei‐Adjei:** formal analysis (equal), investigation (equal), methodology (equal), validation (equal), visualization (equal), writing – original draft (equal). **Jacob Alhassan Hamidu:** formal analysis (equal), investigation (equal), supervision (equal), validation (equal), visualization (equal), writing – original draft (equal). **Benjamin Adjei‐Mensah:** data curation (equal), validation (equal), writing – original draft (equal), writing – review and editing (equal). **Priscilla Nyameyie Ama Hanson:** methodology (equal), resources (equal), software (equal), supervision (equal). **Goodman Kantanka Sarfo:** conceptualization (equal), methodology (equal), supervision (equal), validation (equal), writing – review and editing (equal). **Armstrong Donkoh:** conceptualization (equal), methodology (equal), supervision (equal), validation (equal), writing – review and editing (equal).

## Conflicts of Interest

The authors declare no conflicts of interest.

## Data Availability

All data used is available and can be provided upon reasonable request.
